# Oral candidiasis and cigarette, tobacco, alcohol, and opium consumption in Rafsanjan, a region in the southeast of Iran

**DOI:** 10.1186/s12903-023-02969-1

**Published:** 2023-05-05

**Authors:** Parvin Khalili, Atekeh Movagharipoor, Farimah Sardari, Fatemeh Movaghari Pour, Zahra Jamali

**Affiliations:** 1 Department of Epidemiology, School of Public Health, Social Determinants of Health Research Center, Rafsanjan University of Medical Sciences, Rafsanjan, Iran; 2 Department of Oral Medicine, Dental School, Rafsanjan University of Medical Sciences, Rafsanjan, Iran; 3 Department of Comprehensive Dentistry, School of Dentistry, University of Texas Health San Antonio, 78229-3900 San Antonio, TX USA; 4 Non-Communicable Diseases Research Center, Rafsanjan University of Medical Sciences, Rafsanjan, Iran; 5 Clinical Research Development Unit (CRDU), Niknafs Hospital, Rafsanjan University of Medical Sciences, Rafsanjan, Iran

**Keywords:** Oral candidiasis, Cigarette, Opium, Tobacco, Alcohol, Prospective Epidemiological Research Studies in IrAN (PERSIAN)

## Abstract

**Background:**

We investigated the association between oral candidiasis prevalence and cigarette, tobacco, alcohol, and opium consumption in Rafsanjan, a region in the southeast of Iran.

**Methods:**

This cross-sectional study was conducted using the data of Oral Health Branch of Rafsanjan Cohort Study (OHBRCS) as a part of the Rafsanjan Cohort Study (RCS). RCS included in Prospective Epidemiological Research Studies in IrAN (PERSIAN) was begun in 2015 in the Rafsanjan. A full-mouth examination was done by trained dental specialists. Oral candidiasis was diagnosed based on clinical examination. Information about cigarette, tobacco, and opium smoking and alcohol consumption were collected based on data from self-reported questionaries. Univariate and multivariate dichotomous logistics regression were used to assess the association between oral candidiasis and cigarette, tobacco, alcohol, and opium consumption.

**Results:**

Among 8682 participants with mean age of 49.94 years, the prevalence of oral candidiasis was 7.94%. There was a direct association between cigarette smoking in current and former cigarette smokers with an increased odds of oral candidiasis (OR: 3.26, 95% CI: 2.46–4.33 and OR: 1.63, 95% CI: 1.18–2.25 respectively) in fully adjusted models. There was a dose-response relationship between the odds of oral candidiasis and dose (OR: 3.31, 95% CI: 2.38–4.60), duration (OR: 2.48, 95% CI: 2.04–3.95) and number (OR: 3.01, 95% CI: 2.02–4.50) of cigarette smoking in the 4th quartile compared to reference group.

**Conclusions:**

A dose-response relationship was shown between cigarette smoking and increased odds of oral candidiasis.

## Introduction

Candida is a group of fungi that inhabit the oral cavity in healthy subjects without any specific harm. However, a fungal infection develops when an imbalance in the normal oral flora occurs [1]. Candidiasis is the most common fungal infection in humans which has been reported in the oral cavity of 54.0-71.4% of healthy people [2]. As shown by research, candidiasis is capable of producing nitrosamine and carcinogenic chemical substances which probably play a role in promoting oral cancer. So, the presence of candidiasis should be considered critical as a possible contributor to oral cancer [3].

In previous studies, older age, female gender, use of dentures, the habits of smoking, and diabetes have been associated with an increased risk for oral candidiasis [1, 2, 4]. Correlation between opium consumption, as an immune system suppressor, and candidiasis was investigated in some studies which reported a higher risk of candidiasis in opium users [2, 5, 6]. However, Kathwate’s study has shown a reverse association [7]. Although several previous studies have reported that cigarette smoking [4, 8, 9], tobacco [10], and alcohol [6] were associated with increased oral candidiasis, other researches have not found this association [10, 11].

Consumption of cigarettes and opium remains to be a significant problem for public health. Opium is traditionally used in Iran due to the easy access to this substance and disbelief about its preventive role in diabetes and dyslipidemia [12]. It has been reported that 25.45% and 23.81% of adults of Rafsanjan use cigarettes and opium respectively [13] .

Considering the high prevalence of cigarettes and opium consumption in Rafsanjan [13] and on the other hand, the inconsistent results about the association between smoking and oral candidiasis [4, 6, 8–11], we aimed to investigate the association between oral candidiasis prevalence and cigarette, tobacco, alcohol and opium consumption with a larger sample size in the population of Oral Health Branch of Rafsanjan Cohort Study (OHBRCS).

## Materials and methods

### Study participants

This cross-sectional study was conducted using the data of Oral Health Branch of Rafsanjan Cohort Study (OHBRCS). OHBRCS is a part of the Rafsanjan Cohort Study (RCS) that was designed to investigate the dental and oral health of the participants. RCS as a part of Prospective Epidemiological Research Studies in IrAN (PERSIAN) [14] was begun in 2015 in the Rafsanjan, a region in the southeast of Iran. RCS recruited 10,000 subjects of both genders aged 35–70 years [13]. All subjects signed written informed consent. The oral examination was also a part of RCS routine data collection. About 8682 subjects of the RCS adults participated in the OHBRCS and included in the present study.

### Data collection

Participants were interviewed using a validated, standardized, and detailed questionnaire [13, 14]. The questionnaire contained demographic and anthropometric data, socioeconomic status, medical history, and personal habits. The wealth score index (WSI) was used to measure the Socio-Economic Status (SES) of subjects. Cigarette, tobacco, and opium smoking and alcohol consumption were self-reported. Tobacco use was defined as using naas, hookah, pipe, or chopogh once a week for at least six months. Nass is the name of an addictive herbal medicine that is made from tobacco leaves and is placed in the labial vestibule, and after creating a euphoric effect, it is thrown out. Chopogh and pipe are wooden tools for using tobacco smoke. Hookah is the name of a device for consuming tobacco in which tobacco smoke enters the lungs after passing through water.

Alcohol drinkers were defined as participants who reported drinking approximately 200 ml of beer or 45 ml of liquor, once a week for at least six months [14]. In terms of smoking, participants were divided into categories as nonsmokers, current smokers, and former smokers. The number of cigarette consumption was defined as the average number of times the participant smokes in 24 h. Duration of cigarette consumption included the number of years the participant used cigarettes throughout the participant’s life. The cigarette dose specified the dose of cigarette use throughout the participant’s life (pack-year) [14].

Opium user was defined as a participant that reported opium consumption at least once a week during six months. Opium use duration was defined as the number of years the subject used opium throughout the subject’s life. The opium dose included the dose of opium consumption throughout the subject’s life (dose-year: the number of years the subject used opium once per day). Route of opium consumption included oral or smoking [15]. Duration and dose of opium or cigarette consumption and the number of cigarette consumption were categorized based on quartiles to test for dose-response association.

### Oral data collection

Oral health data was collected through interview and examination. Oral health data included such items as oral hygiene, oral diseases, oral health status, and dental visits. From these variables, in the preset study we analyzed the following items: brushing, using denture, and candidiasis.

A full-mouth examination was done by trained dental specialists. A trained assistant who accompanied the dental specialists recorded the data. Three oral medicine specialist, one periodontist and one general dentist were trained during 2 training sessions for proper diagnosis of oral diseases. Diagnosis of the oral candidiasis was performed based on clinical examination. No laboratory test were utilized [16]. After the diagnosis of the lesion by the examiners, the final diagnosis was approved by three oral medicine specialists. Instruments were unified for all examiners and included dental mirror, dental sound and gauze for scrub. Participants with removable partial or complete dentures were considered as removable denture wearers.

The present study was approved by the Ethics Committee of Rafsanjan University of Medical Sciences (Ethical codes: ID: IR.RUMS.REC.1398.068). Furthermore, guidelines for the report of observational studies in epidemiology (STROBE) were considered and used for performing this study.

### Statistical analyses

Frequency (%) for categorical variables and mean along with the standard deviation (SD) for the quantitative variables were calculated. Baseline characteristics were compared across the groups of the present study using chi-square (χ²) for the categorical variables and a t-test for the continuous variables.

In addition, univariate and multivariate dichotomous logistics regression analyses were used to determine the odds ratios (ORs) and the corresponding 95% confidence intervals (CI) for the relation of cigarette, opium, and alcohol consumption with oral candidiasis in study participants. Four models were used in the regression analysis. Variables with a p value below 0.25 in bivariate analysis were included in the regression models as potential confounder. Potential confounding variables were sequentially entered into models according to their hypothesized strengths of association with candidiasis, cigarette, opium, and alcohol consumption. The baseline model (crude model) was stratified on the condition of cigarette, opium, and alcohol consumption. The adjusted model 1 was adjusted for confounding variables including age (continuous variable), gender (male/ female), education years (continuous variable), wealth status index (continuous variable), body mass index (BMI) (continuous variable), diabetes (yes/no), and hypertension (yes/no). In the adjusted model 2, additional adjustments for use of removable dentures (yes/no) and tooth brushing (yes/no) were added. The adjusted model 3 included additional adjustments for alcohol, opium and cigarette consumption. In the model related to cigarette smoking and candidiasis, the effects of alcohol and opium was controlled and in the model related to opium consumption and candidiasis, the effects of alcohol and cigarette smoking was controlled. Also in the model related to alcohol drinking and candidiasis, the effects of cigarette smoking and opium was controlled.

## Results

Table 1 shows the characteristics of participants including demographic, SES, alcohol, cigarette, tobacco and opium consumption, medical history, and oral health according to oral candidiasis status. In the present study, 4661(53.69%) women and 4021(46.31%) men, with a mean age of 49.94 ± 9.51 were included. The prevalence of candidiasis in all participants was 7.94%. There were significant differences regarding age, gender, education, WSI, BMI, history of diabetes and hypertension, tooth brushing, and wearing dentures among candidiasis and non- candidiasis participants. Compared to the non- candidiasis group, participants with candidiasis were more likely to be older, male, have a denture, and have a history of diabetes or hypertension. Besides, they presented lower education levels, WSI, and BMI and were less likely to brush their teeth (Table 1).

**Table 1:** Characteristics of the study population, overall and according to the candidiasis status among a sample of Rafsanjan population (n = 8682)

Characteristics	Overall (n = 8682)	Without candidiasis (n = 7993)	Candidiasis (n = 689)	P-Value
**Age- yr. no (%)**				< 0.001
35–45	3217(37.06)	3130(31.16)	87(12.63)	
46–55	2697(31.07)	2486(31.11)	211(30.62)	
≥ 56	2767(31.87)	2376(29.73)	391(56.75)	
Mean ± SD	49.94 ± 9.51	49.42 ± 9.44	55.93 ± 8.20	< 0.001
**Gender- no (%)**				< 0.001
Female	4661(53.69)	4415(55.24)	246(35.70)	
Male	4021(46.31)	3578(44.760	443(64.300	
**Education- no (%)**				< 0.001
< 5	2968(34.22)	2634(32.98)	334(48.55)	
6–12	4234(48.81)	3939(49.32)	295(42.88)	
> 13	1472(16.97)	1413(17.69)	59(8.58)	
Mean ± SD	8.64 ± 5.03	8.82 ± 0.01	6.65 ± 4.86	< 0.001
**BMI**				
Mean ± SD	27.86 ± 4.84	27.96 ± 4.79	26.74 ± 5.34	< 0.001
**Wealth score index - no (%)**				
Low	1921(22.16)	1725(21.16)	196(28.49)	< 0.001
Low-middle	2485(28.67)	2243(28.10)	242(35.17)	
Middle-high	2475(28.55)	2303(28.86)	172(25.00)	
High	1788(20.63)	1710(21.43)	78(11.34)	
Mean ± SD	0.04 ± 0.98	0.06 ± 0.98	-0.21 ± 0.97	< 0.001
**Tooth brushing - no (%)**				< 0.001
Yes	6159(70.94)	5767(73.40)	292(42.38)	
NO	2523(29.06)	2126(26.60)	397(57.62)	
**Hypertension- no (%)**				0.030
Yes	1946(22.52)	1769(22.24)	177(25.84)	
No	6694(77.48)	6186(77.760	508(74.16)	
**Diabetes- no (%)**				< 0.001
Yes	1698(19.65)	1521(19.12)	177(25.84)	
No	6942(80.35)	6434(80.88)	508(74.16)	
**Denture - no (%)**				< 0.001
Yes	2859(33.85)	2229(28.68)	630(93.33)	
No	5588(66.15)	5543(71.32)	45(6.67)	

Abbreviations: Body mass index (BMI)

Table 2 shows the personal habits of participants including alcohol, cigarette, tobacco and opium consumption according to oral candidiasis status. There were significant differences regarding alcohol, cigarette and opium consumption among candidiasis and non- candidiasis participants. Compared to the non- candidiasis group, participants with candidiasis were more likely to be alcohol drinkers, cigarette smokers and opium users.

**Table 2:** Personal habits of the study population, overall and according to the candidiasis status among a sample of Rafsanjan population (n = 8682)

Characteristics	Overall (n = 8682)	Without candidiasis (n = 7993)	Candidiasis (n = 689)	P-Value
**Alcohol consumption- no (%)**				< 0.001
Yes	857(9.93)	751(9.45)	106(15.540	
No	7770(90.07)	7194(90.550	576(84.46)	
**Tobacco smoking - no (%)**				0.954
Yes	891(10.33)	821(10.33)	70(10.26)	
No	7736(89.67)	7124(89.67)	612(89.74)	
**Cigarette smoking-no (%)**				< 0.001
Yes	1396(16.18)	1119(14.08)	277(40.62)	
Former	750(8.69)	655(8.24)	95(13.93)	
No	6481(75.12)	6171(77.67)	310(45.45)	
**Dose of cigarette consumption- no (%)**				< 0.001
Non-user	6480(75.30)	6170(77.83)	310(45.72)	
≤ 3.15	533(6.19)	488(6.16)	45(6.64)	
3.16–13.99	553(6.43)	492(6.21)	61(9.00)	
14-26.25	513(5.96)	410(5.17)	103(15.19)	
> 26.25	527(6.12)	368(4.64)	159(23.45)	
**Duration of cigarette consumption- no (%)**				< 0.001
Non-user	6480(75.30)	6170(77.83)	310(45.72)	
≤ 13	521(6.05)	474(5.98)	47(6.93)	
14–23	532(6.18)	462(5.83)	70(10.32)	
24–33	555(6.45)	447(5.64)	108(15.93)	
> 33	518(6.02)	375(4.73)	143(21.09)	
**Number of cigarette consumption- no (%)**				< 0.001
Non-user	6480(75.14)	6170(77.69)	310(45.45)	
≤ 5	671(7.78)	602(7.58)	69(10.12)	
6–14	401(4.65)	343(4.32)	58(8.50)	
15–20	787(9.13)	610(7.68)	177(25.95)	
> 20	285(3.30)	217(2.73)	68(9.97)	
**Opium consumption- no (%)**				< 0.001
Yes	1954(22.65)	1637(20.60)	317(46.48)	
No	6673(77.35)	6308(79.40)	365(53.52)	
**Route of opium consumption- no (%)**				< 0.001
Non-user	6673(77.35)	6308(79.41)	365(53.44)	
Smoking	1800(20.86)	1515(19.07)	285(41.73)	
Oral	154(1.79)	121(1.52)	33(4.83)	
**Dose of opium consumption- no (%)**				< 0.001
Non-user	6673(77.48)	6308(79.52)	365(53.68)	
≤ 1.37	491(5.70)	448(5.65)	43(6.32)	
1.38–11.43	480(5.57)	412(5.19)	68(10.00)	
11.44-30	538(6.25)	439(5.53)	99(14.56)	
> 30	431(5.00)	326(4.11)	105(15.44)	
**Duration of opium consumption- no (%)**				< 0.001
Non-user	6673(77.36)	6308(79.41)	365(53.52)	
≤ 7	525(6.09)	458(5.77)	67(9.82)	
8–14	470(5.45)	395(4.97)	75(11)	
15–20	499(5.78)	424(5.34)	75(11)	
> 20	459(5.32)	359(4.52)	100(14.66)	

The association of oral candidiasis with cigarette, opium and alcohol consumption using logistic regression models is shown in Table 3. In the crude regression model, the odds of oral candidiasis among current and former cigarette smokers, opium users, and alcohol drinkers were higher than those of non- users. This association persisted in fully adjusted model (adjusted model 3) among current and former cigarette smokers (OR: 3.26, 95% CI: 2.46–4.33 and OR: 1.63, 95% CI: 1.18–2.25 respectively), but not in opium users and alcohol drinkers (OR: 0.93, 95% CI: 0.73–1.19 and OR: 0.98, 95% CI: 0.74–1.31 respectively).

**Table 3:** Association of cigarette, opium and alcohol consumption with candidiasis among a sample of Rafsanjan population (n = 8682)

	Crude model	Adjusted model 1	Adjusted model 2	Adjusted model 3
	OR (95%Ci)^a^	OR(95%Ci)^b^	OR (95%Ci)^c^	OR (95%Ci)^d^
**Cigarette consumption**				
No	1	1	1	1
Current	4.93(4.14–5.86)	4.83(3.81–6.11)	3.16(2.43–4.10)	3.26(2.46–4.33)
Former	2.89(2.26–3.68)	2.29(1.71–3.06)	1.59(1.16–2.17)	1.63(1.18–2.25)
**Dose of cigarette consumption**				
Non-user	1	1	1	1
≤ 3.15	1.84(1.33–2.54)	2.19(1.53–3.13)	2.14(1.45–3.16)	2.16(1.46–3.21)
3.16–13.99	2.47(1.85–3.30)	2.60(1.86–3.62)	1.75(1.22–2.50)	1.78(1.23–2.58)
14-26.25	5.00(3.92–6.38)	4.77(3.53–6.46)	2.62(1.88–3.64)	2.68(1.89–3.80)
> 26.25	8.60(6.91–10.70)	6.19(4.66–8.23)	3.23(2.37–4.39)	3.31(2.38–4.60)
**Duration of cigarette consumption**				
Non-user	1	1	1	1
≤ 13	1.92(1.40–2.65)	2.15(1.51–3.06)	1.91(1.30–2.81)	1.94(1.31–2.87)
14–23	2.95(2.24–3.88)	3.67(2.65–5.08)	2.10(1.48-3.00)	2.14(1.48–3.09)
24–33	4.73(3.73–5.99)	4.38(3.28–5.85)	2.59(1.88–3.56)	2.62(1.87–3.68)
> 33	7.40(5.92–9.25)	4.44(3.13–5.92)	2.77(2.03–3.78)	2.84(2.04–3.95)
**Number of cigarette consumption**				
Non-user	1	1	1	1
≤ 5	2.28(1.74-3.00)	2.42(1.77–3.29)	2.05(1.46–2.87)	2.08(1.47–2.93)
6–14	3.37(2.49–4.55)	3.16(2.23–4.47)	2.09(1.43–3.04)	2.13(1.44–3.14)
15–20	5.78(4.72–7.08)	5.14(3.94–6.69)	2.89(2.16–3.86)	2.95(2.17–4.01)
> 20	6.24(4.64–8.38)	5.14(3.62–7.28)	2.94(2.01–4.30)	3.01(2.02–4.50)
**Opium consumption**				
No	1	1	1	1
Yes	3.35(2.85–3.93)	2.29(1.88–2.79)	1.35(1.08–1.68)	0.93(0.73–1.19)
**Route of opium consumption**				
Non-user	1	1	1	1
Smoking	3.23 (2.74–3.81)	2.28(1.87–2.79)	1.36(1.09–1.70)	0.95(0.74–1.22)
Oral	4.68(3.14–6.98)	2.50(1.63–3.84)	1.29(0.82–2.03)	0.77(0.48–1.25)
**Dose of opium consumption**				
Non-user	1	1	1	1
≤ 1.37	1.66(1.19–2.31)	1.28(0.90–1.82)	1.04(0.71–1.51)	0.81(0.55–1.19)
1.38–11.43	2.85(2.16–3.76)	2.01(1.48–2.73)	1.22(0.88–1.72)	0.83(0.59–1.19)
11.44-30	3.90(3.06–4.97)	2.96(2.24–3.90)	1.49(1.10–2.01)	1.01(0.73–1.41)
> 30	5.57(4.36–7.10)	3.25(2.45–4.32)	1.63(1.19–2.22)	1.09(0.78–1.53)
**Duration of opium consumption**				
Non-user	1	1	1	1
≤ 7	2.50(1.90–3.30)	1.94(1.44–2.62)	1.26(0.91–1.74)	0.92(0.65–1.30)
8–14	3.26(2.49–4.26)	2.69(2.00-3.62)	1.61(1.16–2.26)	1.13(0.79–1.61)
15–20	3.02(2.31–3.95)	2.13(1.58–2.87)	1.09(0.79–1.51)	0.76(0.54–1.07)
> 20	4.86(3.81–6.20)	2.55(1.93–3.37)	1.54(1.14–2.10)	0.98(0.70–1.37)
**Alcohol consumption**				
No	1	1	1	1
Yes	1.76(1.41–2.20)	1.69(1.33–2.17)	1.22(0.93–1.60)	0.98(0.74–1.31)

a The baseline model is not adjusted for other confounders

b The adjusted model 1 is adjusted for confounding variables including age (continuous variable), gender (male/ female), education years (continuous variable), wealth status index (continuous variable), body mass index (continuous variable), diabetes (yes/no) and hypertension (yes/no)

c The adjusted model 2 has additional adjustment for removable denture (yes/no) and brushing frequency (categorical variable)

d The adjusted model 3 included additional adjustments for alcohol, opium and cigarette consumption. In the model related to cigarette smoking and candidiasis, the effects of alcohol and opium was controlled and in the model related to opium consumption and candidiasis, the effects of alcohol and cigarette smoking was controlled. Also in the model related to alcohol drinking and candidiasis, the effects of cigarette smoking and opium was controlled

The results were divided into quartiles of cigarette smoking dose. There was a dose-response relationship between the odds of oral candidiasis and dose of cigarette smoking in the 4th quartile (OR: 3.31, 95% CI: 2.38–4.60) compared to reference group. Also, the results were divided into quartiles of cigarette smoking duration and cigarette smoking number. There was a dose-response relationship between the odds of oral candidiasis and duration (OR: 2.48, 95% CI: 2.04–3.95) and number (OR: 3.01, 95% CI: 2.02–4.50) of cigarette smoking in the 4th quartile compared to reference group (Table 3).

Also, the interaction between opium and cigarette consumption and oral candidiasis was investigated. We found that there was no higher odds of candidiasis in subjects who consume opium and cigarette simultaneously (adjusted OR: 1.12, 95% CI 0.65–1.91).

## Discussion

The aim of the present cross-sectional study was to investigate the association between the prevalence of oral candidiasis and cigarette, tobacco, alcohol, and opium consumption in participants of OHBRCS. This region is located in southeastern of Iran and has a high prevalence of opium usage [13]. The present study would provide basic information for better awareness regarding the prevalence and association of oral candidiasis with cigarette, opium, and alcohol consumption for prevention, early diagnosis, and treatment of this lesion.

According to the findings of the present study, the prevalence of oral candidiasis in all participants was 7.94% which was higher than some previous studies done in India [17], Slovenia [18] and in the north of Iran [19]. It was lower than that in the study of Santiwongkarn et al. [20] and Nabavi et al. [2]. Although in agreement with Faraz’s study, oral candidiasis was more frequent among the male gender [17], there are studies indicating that female subjects have a higher prevalence of oral candidiasis [10]. This variation could be in relation to differences in their age ranges, cultures, lifestyle habits, socioeconomic status, dietary habits, study design, sample size, methodology, environmental factors, and variety in diagnostic criteria for oral candidiasis.

The main finding of the present study was that compared to non-users there was a direct association between cigarette, opium, and alcohol consumption with an increased odds of oral candidiasis even after adjusting for some potential confounding variables such as demographic characteristics, WSI, BMI, and medical history. After adjustment for more potential confounders such as wearing removable dentures, tooth brushing, and personal habits (cigarette, opium, and alcohol consumption), no significant association was observed between opium and alcohol consumption with the odds of oral candidiasis. However, a positive significant association was observed between cigarette consumption and oral candidiasis. Thus, for analyses of associations between cigarette, opium, and alcohol consumption and oral candidiasis, these variables should be considered as potential confounders.

The prevalence of oral candidiasis in current cigarette smokers and former cigarette smokers was almost 3 and 1.5 times respectively, compared with non-cigarette smokers. These findings were similar to those reported by several studies [2, 4, 8, 9]. Although the exact mechanism via which cigarette smoking promotes oral growth is yet to be definitively established, it is known that smoking rises thickness of epithelial cells and changes the functional activity of the keratinocytes which may develop candidiasis growth [21]. A plausible theory suggests that cigarette smoking reduces salivary flow rate and consequently decreases pH of saliva. This acidic environment may increase candidiasis development. Furthermore, smoking may decrease salivary immunoglobulin A (IgA) and impair neutrophil functions which both are in favor of developing candidiasis [22].

It has been reported that 23.81% of the Rafsanjan adults are opium users [13]. The findings of the current study indicate that contrary to cigarette smoking, opium consumption decreased the odds of candidiasis about 7% compared to non-opium users which were not statistically significant. Also, the interaction between opium and cigarette consumption was not associated with higher odds of oral candidiasis (OR: 1.12, 95% CI 0.65–1.91). Hadzic et al. found that candidiasis growth is higher in opium users [6], and in the study of Nabavi in Kerman in the southeast of Iran, the frequency of oral candidiasis was significantly higher among opium users [2]. It has been suggested that impaired immune system caused by opium consumption can develop candidiasis growth [5]. In contrast to these studies, Kathwate et al. reported that tramadol as an opioid agonist had the antifungal role in a certain concentration [7]. However, the current study failed to find a relationship between oral candidiasis and opium use. Due to these conflicting results, the association between opium and oral candidiasis should be further evaluated in the follow-up phase of the study.

Also, according to the findings of the present work, the prevalence of oral candidiasis was lower in alcohol drinkers versus non-alcohol drinkers which was not statistically significant. This was consistent with a previous study in Spain [10]. In contrast, Hadzic et al. concluded that alcohol leads to an increase in oral candidiasis [6]. Since, alcohol consumption is a social stigma in Iran and it is illegal due to religious restrictions, thus, it is possible that some subjects did not answer the alcohol use questionnaire correctly. For this reason, there is a probability of the effect of residual confounding.

Furthermore, the authors of the current study didn’t find any association between tobacco consumption and oral candidiasis which was inconsistent with the study of Sheth et al. in Spain that found tobacco users had elevated levels of candidiasis [10].

Regarding the strengths of the present study, the most prominent ones are the population-based nature of the study with a large sample size and a large amount of information for the exposures (cigarette, tobacco, and opium consumption) and potential confounders (demographic characteristics, oral health factors, and medical history). Furthermore, there are a number of limitations. The most important limitation of the present study is the cross-sectional design, thus we cannot conclude the directionality of obtained results because both outcome and exposure variables were evaluated at the same time. Accordingly, it is suggested that this association be reevaluated in the follow-up phase of this prospective study. Also, it is likely that some people have not answered the opium use questionnaire correctly. Thus, these studies are susceptible to self-reporting and recall biases, which may result in an incidence of bias [23–25]. However, the amount of this bias varies according to the geographical area and the understudy populations [25]. Due to the lower social stigma for opium use in this population, the validity of self-report opium in the Rafsanjan population, especially among the adult population, is relatively high [13, 25].

## Conclusion

Cigarette consumption had a dose-response relationship with increased odds of oral candidiasis, while there was no association between oral candidiasis and opium, alcohol, and tobacco consumption. It is necessary for healthcare managers to raise the level of awareness and health literacy among the populations about the adverse effects of cigarette smoking on oral health and to take effective initiatives to prevent and reduce cigarette smoking.

## Data Availability

The data is not available publicly. However, upon a reasonable request, the data can be obtained from the corresponding author.
